# Supplementary figures and images for: Burden of invasive group B Streptococcus disease in non-pregnant adults: A systematic review and meta-analysis
Source: PLoS One. 2021 Sep 30;16(9):e0258030. doi: 10.1371/journal.pone.0258030 (PMC8483371; doi:10.1371/journal.pone.0258030)

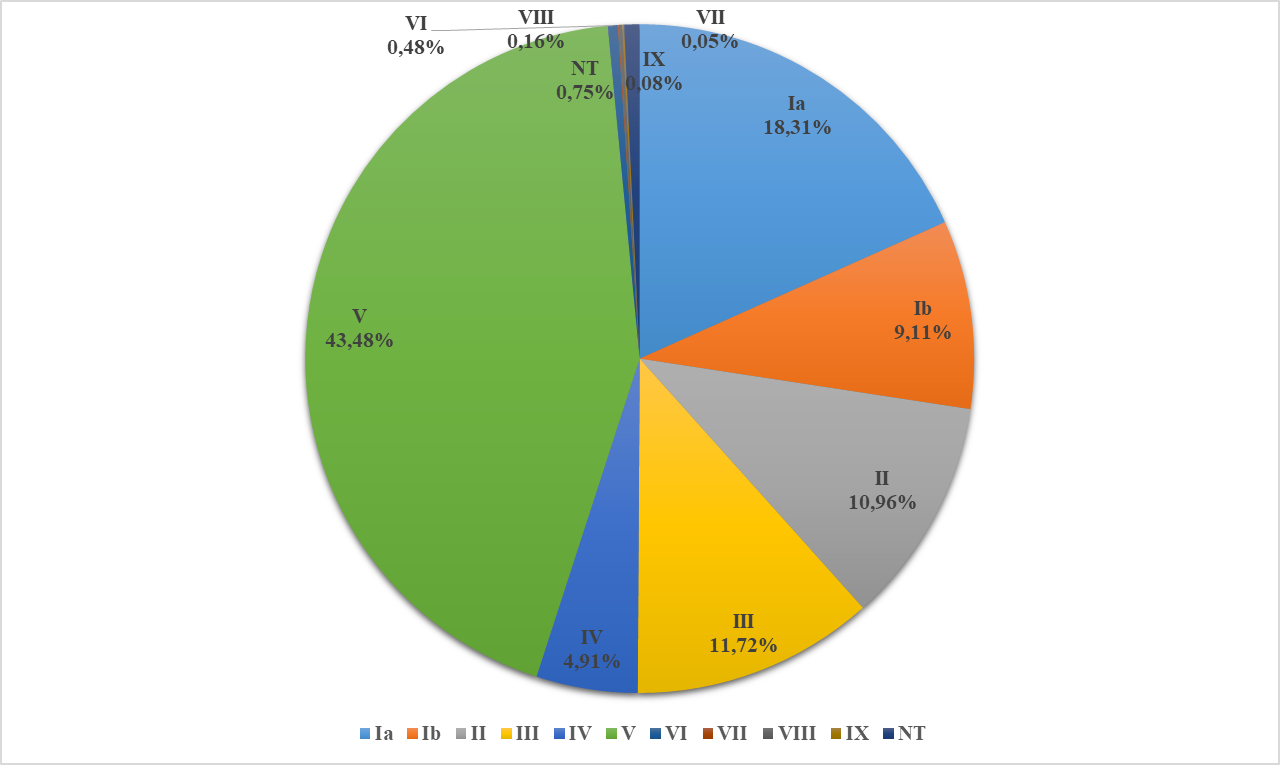

Supplement: S1 Fig — NT = Nontypeable. (TIF) [file pone.0258030.s002.tif]

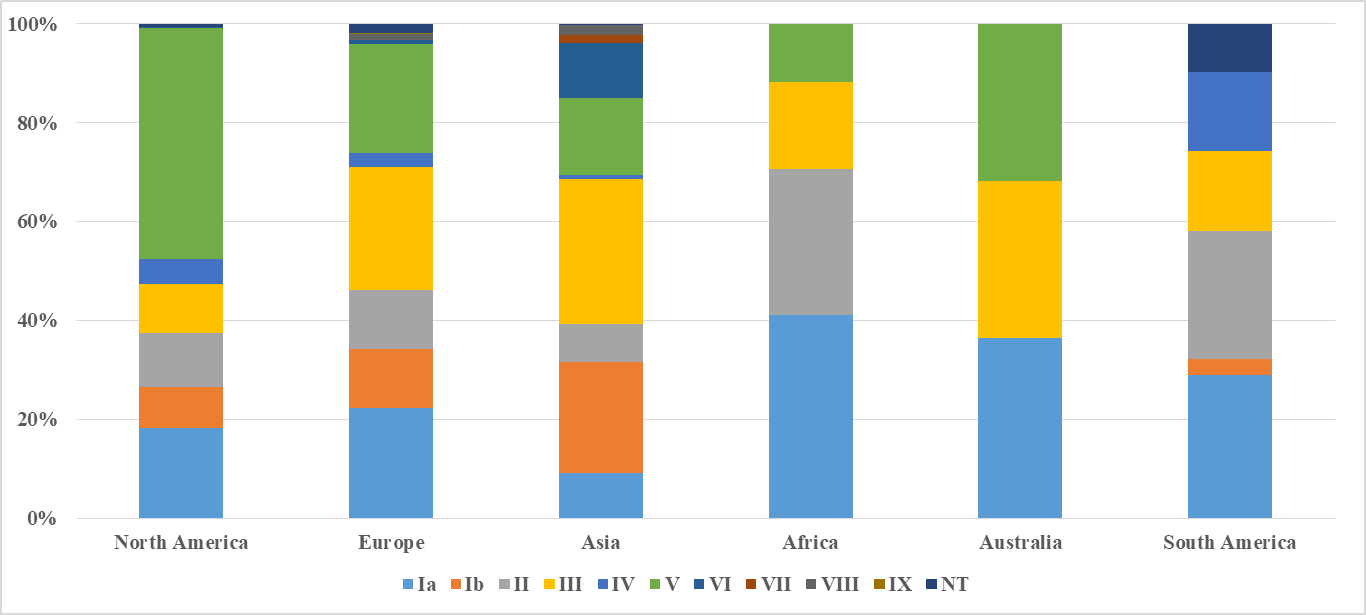

Supplement: S2 Fig — NT = Nontypeable. (TIF) [file pone.0258030.s003.tif]
