# Supplementary material for: Burden of invasive group B Streptococcus disease in non-pregnant adults: A systematic review and meta-analysis
Source: PLoS One. 2021 Sep 30;16(9):e0258030. doi: 10.1371/journal.pone.0258030 (PMC8483371; doi:10.1371/journal.pone.0258030)
Supplement: S1 Table — (DOCX) [file pone.0258030.s004.docx]

**S1 Table. Characteristics of the studies included in the systematic review and meta-analysis**

| **Author** | **Published** | **Year study** | **Country** | **Region** | **Study design** | **Type of surveillance** |
| --- | --- | --- | --- | --- | --- | --- |
| Alhhazmi | 2016 | 2003-2013 | Canada | North-America | Population-based surveillance | Population-based surveillance |
| Barnham | 1989 | 1978-1988 | UK | North-Europe | Population-based surveillance | Laboratory-based surveillance |
| Bjornsdottir | 2016 | 1975-2014 | Iceland | North Europe | Population-based surveillance | Hospital-based surveillance |
| Blumberg | 1996 | 1992-1993 | USA | North-America | Population-based surveillance | Hospital-based surveillance |
| Bolaños | 2001 | 1992-1999 | Spain | South Europe | Population-based surveillance | Laboratory-based surveillance |
| Bunyasontigul | 2011 | 1999-2009 | Thailand | Southeast Asia | Retrospective cohort study | Hospital-based surveillance |
| Camuset | 2015 | 2011 | Réunion Island (France) | Southeast Africa | Prospective hospital-based | Hospital-based surveillance |
| Collin_2020 | 2020 | 2015-2016 | England | North-Europe | Population-based surveillance | Laboratory-based surveillance |
| Cooper | 1998 | 1991-1996 | USA | North-America | Retrospective hospital-based | Hospital-based surveillance |
| Crespo-Ortiz | 2014 | 2004-2012 | Colombia | South America | Retrospective and cross-sectional study | Hospital-based surveillance |
| Darbar | 2007 | 2000-2005 | Australia | Australia | Prospective hospital-based | Hospital-based surveillance |
| Farley | 1993 | 1989-1990 | USA | North-America | Population-based surveillance | Laboratory-based surveillance |
| Francois Watkins | 2019 | 2008-2016 | USA | North-America | Population-based surveillance | Hospital-based surveillance |
| Fujiya | 2019 | 2002-2014 | Japan | Asia | Retrospective cohort study | Hospital-based surveillance |
| Georges | 2008 | 1991-2006 | France | South Europe | Population-based surveillance | Laboratory-based surveillance |
| Gimenez | 1996 | 1983-1993 | Spain | South-Europe | Retrospective study | Hospital-based surveillance |
| Gudjonsdottir | 2015 | 2004-2009 | Sweden | North-Europe | Population-based surveillance | Hospital-based surveillance |
| Huang | 2006 | 2001-2003 | Taiwan | Asia | Retrospective study | Hospital-based surveillance |
| Jenkins | 2010 | 2006-2009 | UK | North-Europe | Population-based surveillance | Hospital-based surveillance |
| Jones | 2006 | 2000-2003 | UK | North-Europe | Prospective study | Hospital-based surveillance |
| Jump | 2019 | 2008-2017 | USA | North-America | Prospective cohort study | Hospital & laboratory-based surveillance |
| Kalimuddin | 2015 | 2011-2015 | Singapore | Southeast Asia | Retrospective cohort study | Hospital-based surveillance |
| Lamagni | 2013 | 1991-2010 | UK | North-Europe | Retrospective study | Laboratory-based surveillance |
| Lambertsen | 2010 | 1999-2004 | Denmark | North-Europe | Population-based surveillance | Laboratory-based surveillance |
| Lee | 2005 | 1991-1999 | Taiwan | Southeast Asia | Prospective study | Hospital-based surveillance |
| Lopardo | 2003 | 1998-1999 | Argentina | South-America | Prospective study | Hospital-based surveillance |
| Matsubara | 2009 | 1998-2007 | Japan | Asia | Retrospective study | Hospital-based surveillance |
| Morozumi | 2016 | 2010-2013 | Japan | Asia | Prospective study | Hospital-based surveillance |
| Mosites | 2019 | 2002-2015 | Canada | North-America | Retrospective study | Laboratory-based surveillance |
| Perovic | 1999 | 1995-1997 | South Africa | Africa | Retrospective study | Hospital-based surveillance |
| Phares | 2008 | 1999-2005 | USA | North-America | Population-based surveillance | Laboratory-based surveillance |
| Ruppen | 2017 | 1998-2015 | Switzerland | Central Europe | Retrospective study | Hospital-based surveillance |
| Schrag | 2000 | 1993-1998 | USA | North-America | Population-based surveillance | Laboratory-based surveillance |
| Schwartz | 1991 | 1982-1983 | USA | North-America | Retrospective population-based surveillance | Laboratory-based surveillance |
| Shelburne | 2013 | 2000-2011 | USA | North-America | Retrospective study | Hospital-based surveillance |
| Skoff | 2009 | 2007 | USA | North-America | Population-based surveillance | Laboratory-based surveillance |
| Slotved | 2020 | 2005-2018 | Denmark | North-Europe | Population-based surveillance | Laboratory-based surveillance |
| Tyrrell | 2000 | 1996 | Canada | North-America | Population-based surveillance | Laboratory-based surveillance |
| Wilder-Smith | 2000 | 1998 | Singapore & Hong Kong | Asia | Prospective study | Hospital-based surveillance |
