# Supplementary material for: Burden of invasive group B Streptococcus disease in non-pregnant adults: A systematic review and meta-analysis
Source: PLoS One. 2021 Sep 30;16(9):e0258030. doi: 10.1371/journal.pone.0258030 (PMC8483371; doi:10.1371/journal.pone.0258030)
Supplement: S2 Table — NA = Not available. (DOCX) [file pone.0258030.s005.docx]

**S2 Table. Participants´ characteristics**

| **Author** | **Age range (years)** | **Age median**  **(years)** | **Age mean**  **(years)** | **Sex** | **Clinical presentation** | **Concomitant disease** |
| --- | --- | --- | --- | --- | --- | --- |
| Alhhazmi | 91 days to 14 years; 15 to > 50 years | NA | NA | NA | NA | NA |
| Barnham | 25-86 | NA | NA | NA | Bacteraemia | Urethral stricture, peripheral vascular disease, ulceration of foot |
| Bjornsdottir | 19-90 | 65.5 | NA | Female 85.57% | Bacteraemia, one joint infection | NA |
| Blumberg | 39.1-75.3 | NA | 57.2 | Female 47% | NA | NA |
| Bolaños | 21-100 | NA | 63 | Female 56% | Bacteraemia mainly, peritoneal fluid, synovial fluid, abdominal abscesses, meningitis | Diabetes 63%, malignancies 22%; liver cirrhosis 19%; history of surgery 12% |
| Bunyasontigul | 15.3-91.6 | NA | 60.1 | Female 59.4% | Bacteraemia | Diabetes 34.6%; high blood pressure 34.6%; cardiovascular disease (CVD) 24.8% |
| Camuset | 25-93 | 59 | NA | Female 50% | Osteoarticular, orthopaedic prosthesis, chronic prostatitis | Type 2 diabetes 63.6%; obesity; cancer; neurogenic bladder |
| Collin_2020 | ≥ 15 | NA | NA |  |  |  |
| Cooper |  | NA | NA | Female 54.5% | Bacteraemia | CVD 35.6%; diabetes 35.6%; cirrhosis 18.6%; malignancy 18.6% |
| Crespo-Ortiz | 17-83 | NA | NA | Female 36,8 | Bacteraemia 59.7% | Diabetes 28%; cancer 24.6%; haematological malignancies 12.3% |
| Darbar | 47.3-78.7 | NA | 63 | NA | Bacteraemia | Diabetes (39 patients); malignancy (19 patients); renal failure (8 patients); cirrhosis (2 patients) |
| Farley | 18-99 | NA | 62 | NA | Bacteraemia, oesteomyelitis, sepsis, peritonitis, pneumonia |  |
| Francois Watkins | ≥18 | NA | NA | NA | NA | Diabetes, CVD, cancer, chronic skin disease |
| Fujiya | 24-91 | 65.5 | NA | Female 56% | Bacteraemia, septic arthritis, skin/soft tissue | Neurological disorders, cirrhosis, chronic kidney disease |
| Georges |  | NA | NA |  | Bacteraemia, meningitis | |
| Gimenez | 40-80 | NA | 62 | Female 46% | Bacteraemia, arthritis, pyelonephritis, peritonitis | Cirrhosis, malignancies, diabetes |
| Gudjonsdottir | 23-103 | 73 | NA | Female 46.4% | Sepsis, erysipelas, pneumonia, endocarditis, meningitis | CVD, diabetes, malignancies |
| Huang | 22-89 | NA | 64.7 | Female 51.1% | Bacteraemia, soft tissue infection | Malignancy, diabetes, liver cirrhosis |
| Jenkins | 28-84 | NA | 63.8 | Female 47% | Prosthetic infections, osteomyelitis | Diabetes 47% |
| Jones | > 60 | NA | NA | NA | Bacteraemia 74.3%; osteoarticular infection 18.6% | |
| Jump | ≥18 | NA | 66.5 | Female 2.9% | Osteomyelitis, bacteraemia without focus, skin of soft-tissue infection, pneumonia or empyema, joint infection, endocarditis, peritonitis, necrotising fasciitis, meningitis | Diabetes 65.0%; obesity 51.6%; chronic heart conditions 31.6%; renal disease 27.6%; chronic pulmonary disease 27.4%; peripheral vascular disease 23.7%; cancer 22.2%; cerebrovascular disease 16.1%; liver disease 14.9%; paralysis 6.5%; dementia 4.3%; peptic ulcer disease 3.9%; rheumatic disease 2.4%; AIDS/HIV 1.3% |
| Kalimuddin | Non-pregnant adults | NA | NA | Female 42.2% | Bacteraemia without focus 23.3%; native joint septic 14%; meningitis 7.1%; skin//soft tissue infection 33.6% | Diabetes 45.3%; high blood pressure 57.1%; hyperlipidaemia 49.5% |
| Lamagni | >90 days to ≥75 years | NA | NA | NA | Bacteraemia 97.2%; joint infection 4%; meningitis 0.9% |  |
| Lambertsen | 16-99 | NA | 62.8 | Female 53% | Bacteraemia, meningitis | Diabetes 15%; alcohol abuse 12%; cancer 7% |
| Lee | 18-85 | NA | 55.5 | Female 31% | Bacteraemia, other | Diabetes 54.9%; malignancies 15.5%; use glucocorticoids 5.6% |
| Lopardo | 21-83 | NA | 58.8 | Female 35.8% | Skin and soft tissues, bones, joints, blood, central nervous system, pleura, abdominal cavity, and kidney infections | Diabetes 22.6%; tumours 12.9% |
| Matsubara | 29-90 | NA | NA | NA | Bacteraemia with and without focus, intraabdominal  infection, meningitis, deep neck infection | |
| Morozumi | 18- >90 | 73.0 | 70.4 | Female 44.7% | Bacteraemia without focus 49.9%; cellulitis 12.9%; pneumonia 9%; endocarditis 3.8%; meningitis 3.8% | Diabetes 30.2%; cancer 24.6%; liver or renal dysfunction 23.3%; cardiac diseases 15.8% |
| Mosites | ≥18 | NA | 53 | Female 33% | Alcohol abuse, bacteraemia, pneumonia, cellulitis | |
| Perovic | 22-87 | NA | 45.6 | Female 52.5% | Bacteraemia 50%; pus at surgery 37.5% (soft tissue abscess 50%, pneumonia 20%, meningitis 7.5%, peritonitis) | Diabetes 27.5%; trauma 25%; HIV 12,5%; alcoholic liver cirrhosis 10% |
| Phares | 0 days - ≥65 | NA | NA | NA | Bacteraemia without focus 48%, bacteraemic cellulitis 22%; pneumonia 11%; osteomyelitis 9%; arthritis 9%; peritonitis 3%; abscess 3% | Diabetes 41%; heart disease 36%; malignancy 17%. Other common factors associated with cases among adults included smoking, obesity, neurologic disorders, renal disease, immunosuppressive conditions, liver disease, and lung disease. |
| Ruppen | ≥ 65 | NA | NA | Female 47% | Bacteraemia 46%, osteomyelitis, prosthetic joint infection, orthopaedic device-associated infection | High blood pressure 63%; diabetes 39% |
| Schrag | 1 - ≥ 65 | NA | NA | NA | bacteraemia 84%; meningitis 4%; synovial fluid 4%; surgical specimen, pleural fluid | Diabetes 37%; CVD 23%; non-haematological cancer 19%; congestive heart failure 15%; alcoholism 11%; cirrhosis 8% |
| Schwartz | ≥ 20 | NA | 59 | NA | Bacteraemia 71%; soft tissue infection 16%; joint fluid infection 9% | Diabetes 29%; CVD 25%; cancer 20%; COPD 16%; alcoholism 14% |
| Shelburne | 27-86 | NA | 56 | Female 56% | Bacteraemia | Malignancies 100%; diabetes 16% |
| Skoff | 18-105 | 62 | NA | Female 40.8% | Bacteraemia without focus 39,3%; skin and soft tissue 25.5%; pneumonia 12.5%; bone infection 9.4%; joint infection 7.8% | Diabetes 44.4%; CVD 21.0%; obesity 16.8%; cancer 14.8%; renal disease 13.2% |
| Slotved | 0-100.7 | NA | NA | **NA** | Bacteraemia, meningitis, other sterile sites´infections |  |
| Tyrrell | ≥ 15 | NA | NA | Female 51.6% | Soft-tissue infections (necrotic tissues, abscesses, cellulitis, ulcers, and wounds), bacteraemia without focus, arthritis/bursitis, and pneumonia | Diabetes, residence in a nursing home, hospitalization, and cancer |
| Wilder-Smith | 24-63 | NA | 45 | Female 63.6% | Meningitis | Diabetes, perforated eardrum, corticoids treatment |

NA= Not available
