# Supplementary material for: Burden of invasive group B Streptococcus disease in non-pregnant adults: A systematic review and meta-analysis
Source: PLoS One. 2021 Sep 30;16(9):e0258030. doi: 10.1371/journal.pone.0258030 (PMC8483371; doi:10.1371/journal.pone.0258030)
Supplement: S3 Table — (DOCX) [file pone.0258030.s006.docx]

**S3 Table. Outcomes of the studies included in the systematic review and meta-analysis**

| **Author** | **No of iGBS cases** | **N (denominator)** | **Incidence** | **No. of deaths** | **Case fatality ratio** |
| --- | --- | --- | --- | --- | --- |
| Alhhazmi | 1,372 | The population of Alberta was 3,134,337 in 2003 and 4,107,762 in 2013 | In 2003, 15-50 years,1.28 cases/100.000; In 2013, 2.84 cases/100,000  In 2003, >50 years, 2.8 cases/100,000; In 2013, 5.99 cases/100,000 population | - | - |
| Barnham | 6 | Catchment area 260,000 population | 0.23 cases per 100,000 population | 2 | 33% |
| Bjornsdottir | 139 | Adult population (>16 years old) 146,267 individuals in 1975 to 254,538 individuals in 2014 | 0 in 1981–82 to 4.41 per 100,000 adults in 2005–06; In 2013–14, 2.17 cases per 100,000 adults; average yearly incidence 1975–1989 of 0.71 cases/100,000 adults | - | - |
| Blumberg | 112 | The population of Atlanta was 2,460,233 during the study period | 5.9 cases in non-pregnant adults/100,000 persons/year | - | - |
| Bolaños | 32 | Catchment area 360,000 population | Annual incidence of 1.5 cases per 100,000 population | - | 31% |
| Bunyasontigul | 101 | - |  | - | 7.9% |
| Camuset | 22 | 217,000 | Overall, 10.1 cases per 100,000 population; in ≥65 years 40.6 cases per 100,000 population | 1 | 4.5% |
| Collin | 2,225 iGBS episodes (2.168 cases) | - | Overall 2.9/100,000 population/year (non-pregnant adults); 15-19 y, 0.33 per 100,000/year; 20-29 y, 1.63 per 100,000/year; 30-39 y, 2.66 per 100,000/year; 40-49 y, 1.87 per 100,000/year; 50-59 y, 2.50 per 100,000/year; 60-69 year, 4.13 per 100,000/year; 70-79 year; 6.76 per 100,000/year; ≥80 y, 15.1 per 100,000/year) | 313 | 12.5% |
| Cooper | 55 |  | 0.31 per 1,000 hospital adult admissions | 9 | 16.4% |
| Crespo-Ortiz | 57 | Catchment area 700,000 population | In period 2004-2012, 0.90 cases per 100,000 population ; In ≥60 years, 0.4 cases per 100,000 population | 10 | 17.5% |
| Darbar | 80 | - |  | 8 | 10% |
| Farley | 140 | - | 4.4 cases per 100,000 population | 30 | 21% |
| Francois Watkins | 21,250 | - | In 2008, 8.1 cases per 100,000; In 2016, 10.9 cases per 100,000 population | 1,381 | 6.5% |
| Fujiya | 52 | - | 2.5 cases per 100,000 | 3 | 5.8% |
| Georges | - | - | In 2006, 15-64 years, 1.8 cases per 100,000; In 1991, >64 years, 4.1 cases per 100,000; In 2006, 9.1 cases/100,000 population | - | - |
| Gimenez | 35 | 600,000 | 0.58 cases per 100,000 population | 2 | 8.7% |
| Gudjonsdottir | 317 | 1,828,140 | 3.47 cases per 100,000 population | 38 | 12% |
| Huang | 94 | - | 0.16 cases/1,000 admissions in 2001; 0.3 cases/1,000 admissions in 2003 | 19 | 20.2% |
| Jenkins | 17 | - | 0.69 cases per 100,000 population | 0 | 0% |
| Jones | 70 | 3,000,000 | 0.78 cases per 100,000 population | - | - |
| Jump | 5,497 | - | In 2008, 9.23 cases per 100,000 person-years; In 2017, 11.67 cases per 100,000 persons-years | 451 | 8.7 % |
| Kalimuddin | 408 | - |  | 30 | 7.4% |
| Lamagni | 13,376 |  | 1-14 years, 0.2 cases per 100,000 population; In adults (>15 years), rates increased from 0.92 to 2.39 cases per 100,000 population | - | - |
| Lambertsen | 411 | - | In 1999, 2.2 cases per 100,000 adults; In 2004, 3.2 cases per 100,000 adults | 58 | 14% |
| Lee | 71 | - | 4.7 cases per 10,000 admissions | 5 | 7% |
| Lopardo | 31 | - | - | 4 | 12.9% |
| Matsubara | 52 | Catchment area 500,000 population | - | 7 | 13.46% |
| Morozumi | 443 | - | - | 45 | 10.2% |
| Mosites | 6 | - | 44.2 cases per100,000 persons-years | 2 | 33% |
| Perovic | 40 | Catchment area 3,000,000 population | - | 14 | 35% |
| Phares | 11,663 adult non-pregnant cases [15-64 years 6,087 cases; ≥65 years 5,576 cases] | - | In 2005, 15-64 years, 3.4 in 1999 to 5.0 per 100,000 population; ≥65 years, from 21.5 in 1999 to 26.0 per 100,000 in 2005; Overall adult, 6.0 in 1999 to 7.9 cases per 100,000 population in 2005 corresponding to 6.95 per 100,000 population in non-pregnant adults | 1,202 | 10.3% |
| Ruppen | 171 | - |  | 9 | 5% |
| Schrag | 13,200 adult non-pregnant cases [15-64 years, 6,300 cases; ≥65 years, 6,900 cases] | - | 90 days -14 years, 0.2 cases per 100,000 population; ≥15 years, 11.9 cases per 100,000 population; 15-64 years, 3.6 cases per 100.000; ≥65 years, 20.1 cases per 100,000 population | 589 deaths | 11.5% |
| Schwartz | 56 | - | 2.4 cases per 100,000 population | - | - |
| Shelburne | 147 | - |  | 9 | 6% |
| Skoff | 1,546 | - | 7.3 cases per 100,000 population | 114 | 7.5% |
| Slotved |  | - | 5-19 years, 0.08 cases per 100,000; 20-64 years, 1.35 cases per 100,000; 65-74 years, 5.35 cases per 100,000; > 75 years, 9.80 cases per 100,000 population |  |  |
| Tyrrell | 91 | - | 15-19 years, 1.1 cases per 100,000; 20-29 years 0.0 cases per 100,000; 30-39 years, 1.7 cases per 100,000; 40-49 years 3.5 cases per 100,000; 50-64 years, 5.9 cases per 100,000; ≥65 years, 11.9 cases per 100,000 non-pregnant adults.  Overall, 4.1 cases per 100.000 non-pregnant adults | 5 | 5.5% |
| Wilder-Smith | 11 | Catchment area 1,000,000 population |  | 1 | 9.1% |
