# Supplementary material for: Burden of invasive group B Streptococcus disease in non-pregnant adults: A systematic review and meta-analysis
Source: PLoS One. 2021 Sep 30;16(9):e0258030. doi: 10.1371/journal.pone.0258030 (PMC8483371; doi:10.1371/journal.pone.0258030)
Supplement: S4 Table — (DOCX) [file pone.0258030.s007.docx]

**S4 Table. Application of the Johanna Briggs Institute Appraisal Tool for quality assessment**

**Table S4. Application of the Johanna Briggs Institute Appraisal Tool for quality assessment, continued**
