# Supplementary material for: Burden of invasive group B Streptococcus disease in non-pregnant adults: A systematic review and meta-analysis
Source: PLoS One. 2021 Sep 30;16(9):e0258030. doi: 10.1371/journal.pone.0258030 (PMC8483371; doi:10.1371/journal.pone.0258030)
Supplement: S5 Table — PEN Penicillin, ERY Erythromycin, GEN Gentamycin, S Susceptible, AMP Ampicillin, AZM Azithromycin, TCY Tetracycline, R Resistant, CEF Cephalothin, MIN Minocycline, CHL Chloramphenicol, I Intermediate, CTX Cefotaxime, CLI Clindamycin, NS Percentage not specified, CXM Cefuroxime, VAN Vancomycin, * It includes not only adults but also neonates and children, Classification as susceptible, resistant, or intermediate as reported in the study. (DOCX) [file pone.0258030.s008.docx]

**S5 Table. Reported antimicrobial resistance among the selected studies (%)**

| Author | Year study | PEN | AMP | CEF | CTX | CXM | CRO | ERY | AZM | MIN | CLI | VAN | High dose GEN | LVX | TCY | CHL |  |
| --- | --- | --- | --- | --- | --- | --- | --- | --- | --- | --- | --- | --- | --- | --- | --- | --- | --- |
| Bolaños | 1992-1999 | S (100%) |  |  |  |  |  | S (100%) |  |  | S (100%) |  | R (3%) |  |  |  |  |
| Bunyasontigul | 1999-2009 | S (98%) |  | S (100%) |  |  |  | S (99%) |  |  |  |  |  |  |  |  |  |
| Camuset | 2011 | S (100%) |  |  |  |  |  | R (12%) |  |  |  |  |  |  |  |  |  |
| Crespo-Ortiz | 2004-2012 | S (98.6%) |  |  |  |  |  | R (8.9%) |  |  |  |  |  |  |  |  |  |
| Darbar | 2000-2005 | S (100%) |  |  |  |  |  | R (8%) |  |  |  |  |  |  |  |  |  |
| Francois Watkins | 2008-2016 | R (0.50%) |  |  |  |  |  | R (54.8%) |  |  | R (43.2%) |  |  | R (2.3%) | R (83.9%) |  |  |
| Fujiya | 2002-2014 | S (100%) | S (100%) |  | S (100%) |  |  | R (19%) |  |  | R (12%) | S (100%) |  | R (23%) |  |  |  |
| Huang | 2001-2003 | S (98%) |  |  |  | S (100%) | S (100%) |  |  |  |  | S (100%) |  |  |  |  |  |
| Jenkins | 2006-2009 | S (100%) |  |  |  |  |  | I/R (35.3%) |  |  |  | S (100%) |  |  |  |  |  |
| Lamagni* | 1991-2010 | S (100%) |  |  |  |  |  | R (15%) |  |  | R (9%) |  |  |  |  |  |  |
| Lee | 1991-1999 | S (100%) |  |  | S (100%) |  |  | R (NS) | R (NS) |  | R (NS) |  |  |  | R (95%) | R (NS) |  |
| Lopardo | 1998-1999 | S (100%) |  |  |  |  | S (100%) | R (5.2%) | R (5.2%) | R (69.0%) | R (1.7%) |  |  |  | R (72.4%) |  |  |
| Matsubara | 1998-2007 | S (100%) | S (100%) |  | S (100%) |  |  | R (2%) |  |  | R (3%) | S (100%) |  | R (31%) |  |  |  |
| Morozumi | 2010-2013 | S (100%) | S (100%) |  |  |  |  | R (19.2%) |  |  |  |  |  | R (40.2%) |  |  |  |
| Perovic | 1995-1997 | S (100%) |  |  |  |  |  |  |  |  |  |  |  |  |  |  |  |
| Phares* | 1999-2005 | S (100%) | S (100%) |  |  |  |  | R (32%) |  |  | R (15%) | S (100%) |  |  |  |  |  |
| Shelburne | 2000-2011 | S (100%) |  |  | S (100%) |  |  | R (52%) |  |  |  |  |  | S (98%) | R (89%) |  |  |
| Tyrrell | 1996 | S (100%) | S (100%) |  |  |  |  | R (6.7%) |  |  | R (1.1%) | S (100%) |  |  |  |  |  |
| Wilder-Smith | 1998 | S (100%) |  |  |  |  |  |  |  |  |  |  |  |  |  |  |  |
| PEN Penicillin ERY Erythromycin GEN Gentamycin S Susceptible  AMP Ampicillin AZM Azithromycin TCY Tetracyclin R Resistant  CEF Cefalotin MIN MInocyclin CHL Chloramphenicol I Intermediate  CTX Cefotaxime CLI Clindamycin NS Percentage not specifed  CXM Cefuroxime VAN Vancomycin * It includes not only adults but also neonates and children | | | | | | | | | | | | | | | | | |

Note: Classification as susceptible, resistant, or intermediate as reported in the paper.
