# Supplementary material for: Burden of invasive group B Streptococcus disease in non-pregnant adults: A systematic review and meta-analysis
Source: PLoS One. 2021 Sep 30;16(9):e0258030. doi: 10.1371/journal.pone.0258030 (PMC8483371; doi:10.1371/journal.pone.0258030)
Supplement: Attachment — Submitted filename: Response to reviewers.docx [file pone.0258030.s009.docx]

**Response to reviewers**

We would like to thank the reviewers for their comments that undoubtedly have contributed to improve the manuscript.

Reviewer #1: In this systematic review the authors summarize the worldwide incidence and case fatality of invasive group B streptococcal disease in adults (excluding pregnant women). Database searches, quality appraisal and meta-analytical methods have been applied and reported appropriately, although I assume that a supplementary appendix was submitted which was not available for peer review (see comments #2 and #3 below)? The main limitations are duly acknowledged. This is a timely and well-presented systematic review and analysis. Timely because sufficient iGBS studies have accrued and, despite the heterogeneity between study estimates, there does appear to be an increase in incidence, albeit offset by a reduction in case fatality.

1. Abstract has a 95% CI written as "X-Y". Corrected. Page 2, line 55
2. Quality appraisal - a summary of the QA needs to be given in the results and QA ratings provided in a supplementary file. Corrected. QA results in page 13, lines 214-226. Table S4 in Supplementary information file
3. Data extraction - extracted data need to be provided in a supplementary file. Corrected. Supplementary information file provided
4. Results - a statement in the results that between-study heterogeneity was high (>60%) or very high (>90%) in most of the sub-groups would be helpful. Corrected. Statement provided in page 13, line 227
5. Results - meta-regression by time period - the p-value needs to be reported. Corrected. P values provided in page 14, line 244; page 15, line 255
5. Discussion - where heterogeneity is discussed, I would be interested to know whether the authors could identify any possible reasons for major between-study differences in incidence and case-fatality (within a sub-group), e.g. Schrag (2000) vs. Tyrrell (2000) and others (where Schrag reported much higher incidence). Discussed in page 18, lines 335-349
6. Discussion - I assume that a trend was not evident (statistically) because of the heterogeneity. As in, there is an apparent trend (doubling in incidence), but meta-regression won't detect it because of the between-study variation. Yes, thanks.
7. Figures - unfortunately Stata is not the best tool for creating Forest plots (I say that from experience)! Any additional tweaking that could be done, e.g. to report N without the +08 and to remove the sub-group subtotals where I2 and p are blank, would be welcome. I don't know if a newer version of Stata might bring improvements. Thank you for this remark. Figures look much better now!! Corrected. New corrected figures attached (+08 removed and full figures displayed I^2^ and p blank, deleted).

Reviewer #2: The authors conducted a systematic review and meta-analysis of GBS invasive disease in non-pregnant adults. The manuscript is of value to the field, revealing an overall increase in GBS infections as well as of antimicrobial resistance. The manuscript is well done, well written and fluent in all its parts. I have reviewed the paper from the perspective of a microbiologist, and I’m not confident I have the expertise to evaluate the statistical tools of meta-analyses; yet the methodologies used seem suitable for the major objectives proposed and the findings obtained are of value and well discussed. The conclusions are clearly reported and argued. The paper brings new insights into the dynamics of GBS population, contributing to a clearer picture of increasing incidence of non-neonatal group B streptococcal infections.

I have only one very minor comment: In the abstract (line 55), the 95% confidence interval for incidence rate of iGBS in ≥65 years old group is missing. Corrected. Page 2, line 55
